# The role of *JrLACs* in the lignification of walnut endocarp

**DOI:** 10.1186/s12870-021-03280-3

**Published:** 2021-11-03

**Authors:** Pingping Li, Hongxia Wang, Pan Liu, Yaoling Li, Kai Liu, Xiuhong An, Zhihua Zhang, Shugang Zhao

**Affiliations:** 1 College of Life Sciences, Hebei Agricultural University, Baoding, 071001 China; 2 Mountainous Areas Research Institute, Hebei Agricultural University, Baoding, 071001 China; 3 College of Horticulture, Hebei Agricultural University, Baoding, 071001 China; 4 Research Center for Agricultural Engineering Technology of Mountain District of Hebei, Baoding, 071001 China

**Keywords:** Walnut, Laccase, Shell, Lignin

## Abstract

**Background:**

The walnut shell, which is composed of a large number of sclereids originating from the lignified parenchyma of the endocarp, plays an important role in fruit development and during harvesting and storage. The physical and chemical properties of walnut shells are closely related to the lignin content. Laccase is the key enzyme responsible for lignin biosynthesis by the polymerization of monolignols and plays crucial roles in secondary cell wall formation in plants. In this study, we screened and identified laccase family genes from the walnut genome and investigated the expression of laccase during endocarp lignification in walnut.

**Results:**

A total of 37 laccase genes were screened from the walnut genome and distributed on nine chromosomes and classified into 6 subfamilies, among which subfamily IV showed distinct expansion. We observed that endocarp lignification started 44 days after flowering (DAF), and at later periods, the lignin content increased rapidly, with growth peaks at 44–50 DAF and 100–115 DAF. The lignification of the endocarp proceeded from the outside to the inside, as demonstrated by section staining in combination with endocarp staining. Furthermore, the changes in the expression of laccase family genes in the endocarp at different developmental stages were studied, and *JrLACs* showed different expression trends. The expression of nine genes showed significant increase after 44 DAF, and among these, *JrLAC12–1*, *JrLAC12–2* and *JrLAC16* showed a significant change in expression at the lignification stage. A study of the expression of *JrLACs* in different tissues and at various endocarp developmental stages revealed, that most *JrLACs* were expressed at low levels in mature tissues and at high levels in young tissues, in particular, *JrLAC12–1* showed high expression in the young stems. A significant positive correlation was found between the expression of *JrLAC12–1* and the variation in the lignin content in the endocarp.

**Conclusion:**

Laccase genes play an important role in the lignification of the walnut endocarp, and *JrLACs* play different roles during fruit development. This study shows that *JrLAC12–1* may play a key role in the lignification of endocarp.

**Supplementary Information:**

The online version contains supplementary material available at 10.1186/s12870-021-03280-3.

## Background

Walnut (*Juglans regia* L.) is an important oil resource and an economically important forest tree that is widely distributed in many temperate areas of the world, including Asia, the Americas, Europe and Oceania [1]. The walnut fruit consists of a hull, shell (endocarp) and seed (kernel), and the hull and shell together form the pericarp to protect the kernel [2]. The kernel is rich in fatty acids, proteins, and amino acids, among other substances, and is preferred by many people for consumption in various ways, such as in baked goods, salads and jams. For a long time, much attention was given to the quality and nutrition of walnut kernels, and the crucial role of the shell was often ignored. In particular, the shell plays an important role in fruit development [3], during harvest and transport [4, 5], and even during the process of storage [6]. Shell can also be used to make xylose [7], medium [8], activated carbon [9], and extracted pigment [10], which are widely utilized. In particular, a significant correlation exists between the shell structure and quality of walnut kernel [5]. Therefore, the development and structural characteristics of the walnut shell has received increasing attention from scholars [11–15]. The shell is the endocarp of the walnut fruit and consists of sclereids derived from lignification of the parenchyma originating from the primary meristem [13]. Lignification of the parenchyma is the key step in the development of walnut shells. Zhao *et al*. [16] confirmed that the development of walnut shells is closely related to lignin biosynthesis, and lignin is the major factor affecting the shell structure [13].

The process of walnut shell formation involves lignification of the endocarp parenchyma. Lignification refers to the process of the enzyme-catalyzed oxidation-mediated polymerization of three main hydroxycinnamyl alcohols—*p*-coumaryl, coniferyl, and sinapyl alcohols (monolignols)—into lignin, which is then deposited in the secondary cell wall in plant vascular tissues and mechanical tissue [17]. Lignin is a phenylpropanoid-derived polymer that functions as a major component of the secondary cell wall and provides hydrophobicity and protection against pathogens [18, 19]. In plants, a series of enzymes involved in phenylalanine metabolism during cell wall formation have been reported, and most key phenylpropanoid biosynthetic enzymes are also critical for lignin biosynthesis [20]. Phenylalanine is catalytically converted in a stepwise manner to *p*-coumaroyl-CoA by phenylalanine ammonia-lyase (PAL), cinnamate 4-hydroxylase (C4H) and 4-coumarate:CoA ligase (4CL). Subsequently, quinate/shikimate *p*-hydroxycinnamoyltransferase (HCT), *p*-coumaroylshikimate 3′-hydroxylase (C3’H), caffeoyl shikimate esterase (CSE), caffeic acid O-methyltransferase (COMT), caffeoyl-CoA O-methyltransferase (CCoAOMT), cinnamoyl-CoA reductase (CCR), ferulate 5-hydroxylase (F5H) and cinnamyl alcohol dehydrogenase (CAD), which work downstream of 4CL, and the catalyzed reactions provide precursors for all of the downstream metabolites [21]. Ultimately, lignin monomers are converted to lignin polymers by reactions catalyzed by peroxidase (POD) or laccase (LAC) [22], and LAC plays an important role in this process [23].

LAC (EC 1.10.3.2) was first identified from the Japanese lacquer tree (*Rhus vernicifera*) [24] and belongs to a group of three blue multicopper oxidases (LMCOs) belonging to a large gene family found in plants [25], fungi [26] and bacteria [27]. A large number of results have shown that LAC can polymerize lignin monomers to form lignin in various plant tissues [28]. Liang *et al*. [29] introduced a mutation in *AtLAC15* that affected both the extractable lignin and soluble proanthocyanidin content in *Arabidopsis* seeds. *LAC4* and *LAC17* contribute to the constitutive lignification of *Arabidopsis* stems, and *LAC17* is involved in the deposition of G-lignin units in fibers [30]. In the whole genome of pear, 41 LACs have been identified, and among these, *PbLAC1* is involved in lignin biosynthesis and cell wall development, but the lignin content and cell wall thickness are not significantly changed in *PbLAC14*-overexpressing transgenic *Arabidopsis* plants [31]. The promoter of *LAC18* could be significantly activated by the PbrMYB169 protein by binding to AC elements, and the overexpression of *PbrMYB169* increased both lignin deposition and the cell wall thickness [32]. Furthermore, transgenic *Arabidopsis* plants overexpressing *MsLAC1* exhibit an increased G-lignin content, although recombinant *MsLAC1* appears to prefer sinapyl alcohol as a substrate [33].

The role of LAC in plant development has received much attention. Although LACs have been identified and classified in many species, such as *Arabidopsis* [34], *Citrus sinensis* [35], soybean [36], *Setaria viridis* [37], rice [38], sugarcane [39], sweet cherry [40] and cotton (*Gossypium arboreum* and *Gossypium raimondii*) [41], an investigation of the LAC gene family in walnut remains to be conducted. In contrast, the formation of naked kernels, dissilient nuts and stained nuts has become a serious problem due to the rapid increase in thin-shell walnut cultivars in China [5]. In this study, we screened and identified the LAC genes in the walnut genome. In addition, changes in *JrLAC* expression during endocarp lignification were analyzed, and the expression of *JrLAC12–1* and *JrLAC14–5* was found to change significantly during endocarp lignification. *JrLAC12–1* was then used to clarify the role of LACs in endocarp lignification. Therefore, the present study lays the foundation for understanding the function of LACs in endocarp lignification.

## Result

### Identification and analysis of LAC genes in the walnut genome

A hidden Markov model was constructed using the *Arabidopsis* LAC family protein sequence information, and the model was used to search walnut protein sequences for the initial screening of 72 walnut LAC family genes. Based on a Pfam structural domain analysis, 37 walnut LAC family genes were finally obtained after eliminating duplicate redundancy, and these genes were associated with 9 chromosomes and encoded 37 protein sequences. The genes were named according to their annotation information, and the position and structural information of these genes are shown in Table 1. The length of the coding sequence (CDS) ranged from 1671 to 1848 bp. The smallest translated protein was 556 aa, and the largest was 615 aa. The predicted molecular weight (MW) of these proteins ranged from 60.86 kD (JrLAC4–4) to 68.40 kD (JrLAC9–1), and the theoretical isoelectric point (pI) ranged from 4.67 to 9.9. *JrLAC12–1*, which is located on chromosome 7, including 6 exons and 5 introns, had a length of 3981 bp, and the length of the CDS was 1743 bp, encoding 580 amino acids.

**Table 1:** Characteristic of JrLACs family genes from walnut (*J. regia* L.)

Name	Locus ID	Gene Length (bp)	Chromosomes	Exon	Intron	NCBI Reference	CDS (bp)	Amino acid length (aa)	PI	MW (Kd)
*JrLAC1*	108984288	3155	Chr 6	5	4	XM_035690758.1	1749	582	7.08	65.56
*JrLAC2–1*	108983777	3046	Chr 5	6	5	XM_018955536.2	1752	583	9.46	64.34
*JrLAC2–2*	108990223	2839	Chr 4	6	5	XM_018964112.2	1812	603	9.90	66.96
*JrLAC2–3*	109013004	2959	Chr 14	6	5	XM_035685151.1	1770	589	9.79	65.37
*JrLAC4–1*	108994849	2901	Chr 3	6	5	XM_018970242.2	1674	557	9.37	60.93
*JrLAC4–2*	108994944	3025	Chr 3	6	5	XM_018970384.2	1671	556	9.60	61.01
*JrLAC4–3*	109000806	2976	Chr 4	6	5	XM_018977812.2	1674	557	9.23	61.03
*JrLAC4–4*	109021622	3890	Chr 3	6	5	XM_019004281.2	1677	558	8.62	60.86
*JrLAC4–5*	109012365	3736	Chr 14	6	5	XM_035685124.1	1683	560	9.49	61.56
*JrLAC6*	108989255	3022	Chr 7	6	5	XM_018962803.2	1719	572	6.78	63.74
*JrLAC7–1*	108984801	3341	Chr 3	6	5	XM_018956860.2	1719	572	6.19	62.82
*JrLAC7–2*	109002960	3315	Chr 3	6	5	XM_035688863.1	1710	569	6.37	62.82
*JrLAC9–1*	108981170	3632	Chr 15	7	6	XM_018952257.2	1848	615	4.80	68.40
*JrLAC9–2*	109013371	4275	Chr 6	7	6	XM_018995434.2	1773	590	4.70	66.64
*JrLAC9–3*	109011443	4877	Chr 6	7	6	XM_018992655.2	1764	587	4.67	65.80
*JrLAC9–4*	109016897	4352	Chr 6	7	6	XM_035691226.1	1707	568	5.04	63.06
*JrLAC11–1*	108997567	2848	Chr 12	6	5	XM_018973904.2	1680	559	8.51	61.91
*JrLAC11–2*	109013800	2488	Chr 13	6	5	XM_018996016.2	1692	563	8.70	62.67
*JrLAC12–1*	108991934	3981	Chr 7	6	5	XM_018966361.2	1743	580	7.60	63.77
*JrLAC12–2*	109004750	3113	Chr 12	6	5	XM_018983416.2	1743	580	8.96	63.70
*JrLAC13*	108996980	2749	Chr 14	6	5	XM_018973047.2	1728	575	8.27	63.43
*JrLAC14–1*	108983274	3541	Chr 6	7	6	XM_018954858.2	1722	573	8.62	63.56
*JrLAC14–2*	108985628	4451	Chr 6	7	6	XM_018957991.2	1701	566	7.24	62.70
*JrLAC14–3*	108985630	3993	Chr 6	7	6	XM_035691062.1	1716	571	5.84	63.26
*JrLAC14–4*	108991517	3831	Chr 6	7	6	XM_035691227.1	1689	562	5.13	62.27
*JrLAC14–5*	118348788	3844	Chr 6	7	6	XM_035691296.1	1710	569	8.23	63.19
*JrLAC14–6*	109008603	3096	Chr 6	7	6	XM_018988767.2	1704	567	8.57	62.84
*JrLAC14–7*	109013368	3557	Chr 6	7	6	XM_018995430.2	1758	585	4.99	65.53
*JrLAC14–8*	109013369	3789	Chr 6	7	6	XM_018995431.2	1785	594	5.12	66.36
*JrLAC15–1*	109002896	4785	Chr 6	7	6	XM_018980818.2	1785	594	5.39	66.82
*JrLAC15–2*	109014678	3513	Chr 6	6	5	XM_018997175.2	1743	580	6.90	64.68
*JrLAC15–3*	109013370	3947	Chr 6	7	6	XM_018995432.2	1782	593	5.33	66.41
*JrLAC16*	109002717	4202	Chr 12	6	5	XM_035683175.1	1668	555	8. 80	61.21
*JrLAC17–1*	108979069	3052	Chr 5	6	5	XM_018949655.2	1749	582	9.02	64.36
*JrLAC17–2*	108979942	2777	Chr 3	6	5	XM_018950745.2	1773	590	9.23	64.83
*JrLAC17–3*	108989607	3368	Chr 14	6	5	XM_018963281.2	1758	585	9.00	64.46
*JrLAC17–4*	109009009	3406	Chr 14	6	5	XM_018989328.2	1764	587	8.74	64.99

### Analyses of the evolution, gene structure and motif distribution of JrLAC family members

**Fig. 1:**
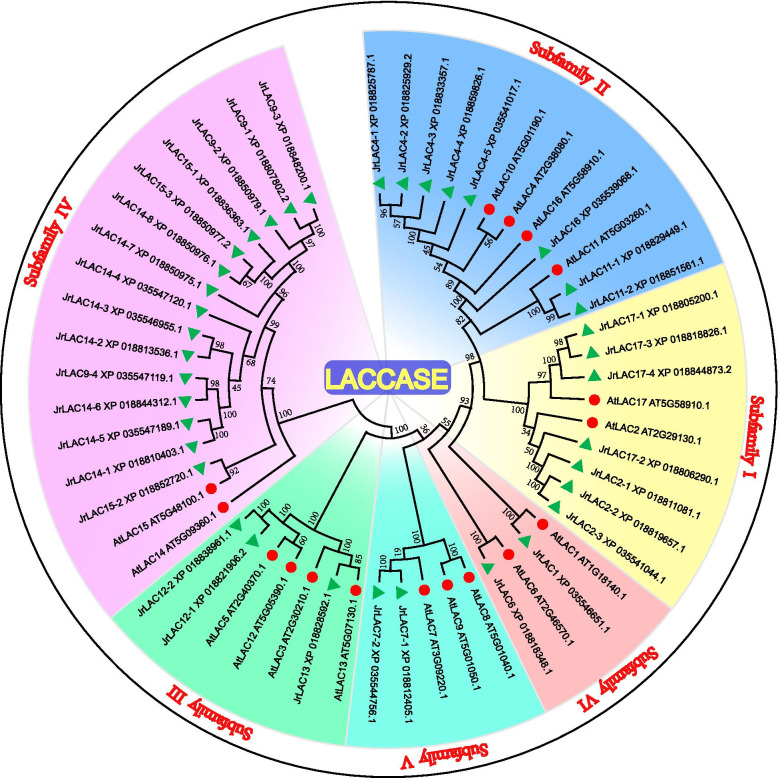
Phylogenetic analysis of *Arabidopsis thaliana* and *Juglans regia* regarding LACs. The areas with different colors represent different subfamilies. The evolutionary history of laccases was created using the maximum likelihood method. The percentage of replicate trees in which the associated taxa clustered together in the bootstrap test (1000 replicates) is shown next to the branches

**Fig. 2:**
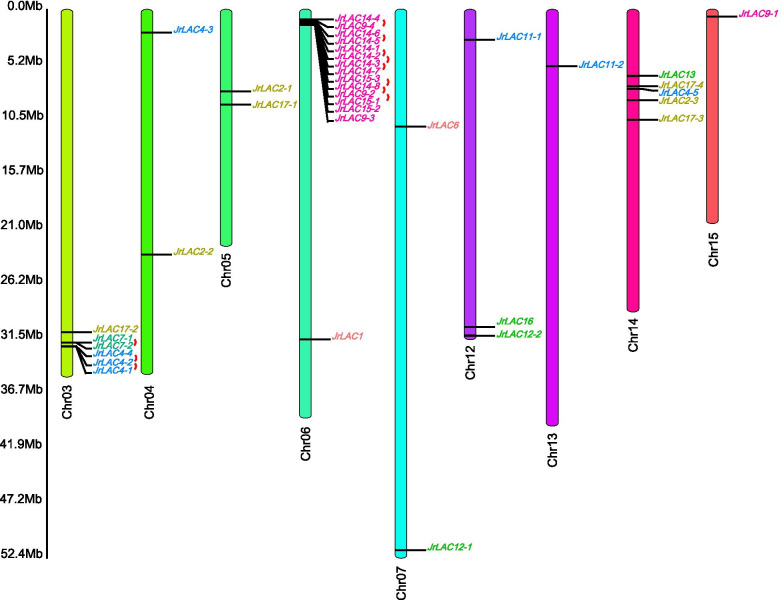
Chromosome distribution of JrLAC genes in walnut. Different line colors represent different chromosomes and marks on them are the corresponding genes and their locations. The small red arc represents pairs of tandem repeated genes

The phylogenetic tree of the walnut LAC protein sequence and the *Arabidopsis* LAC protein sequence was constructed with the maximum likelihood method using MEGA 7.0 software (Fig. 1). The phylogenetic tree indicates that the LAC gene families of *J. regia* and *Arabidopsis thaliana* are similar. According to the classification of *Arabidopsis* LAC genes, the 37 JrLAC genes were classified into 6 subfamilies: I-VI. Among them, subfamily IV was the largest subfamily, with significant gene duplication and expansion, and contained 15 genes: *JrLAC9–1*, *JrLAC9–2*, *JrLAC9–3*, *JrLAC9–4*, *JrLAC14–1*, *JrLAC14–2*, *JrLAC14–3*, *JrLAC14–4*, *JrLAC14–5*, *JrLAC14–6*, *JrLAC14–7*, *JrLAC14–8*, *JrLAC15–1*, *JrLAC15–2* and *JrLAC15–3*. A chromosomal localization analysis of the subfamily IV genes showed that all the genes, with the exception of *JrLAC9–1* (localized on chromosome 15) were located on chromosome 6 and were extremely close together to, form a gene cluster (Fig. 2). Subfamily I contained seven genes, which were mainly localized on chromosomes 3, 4, 5, and 14. Subfamily II contained eight genes, which were mainly localized on chromosomes 3, 4, 12, 13, and 14. Among these genes, *LAC4–1*, *LAC4–2* and *LAC4–4* formed another gene cluster together with *LAC17–2* of subfamily I and *LAC7–1* and *LAC7–2* (the only two genes of subfamily V) on chromosome 3. Subfamily III contained only three JrLAC genes, namely, *JrLAC12–1*, *JrLAC12–2*, and *JrLAC13*, which are localized on chromosomes 7, 12, and 14, respectively. *JrLAC1* and *JrLAC6* belonged to subfamily VI and were localized on chromosomes 6 and 7, respectively. Interestingly, no JrLAC genes were found on chromosomes 1, 2, 8, 9, 11 and 16.

**Fig. 3:**
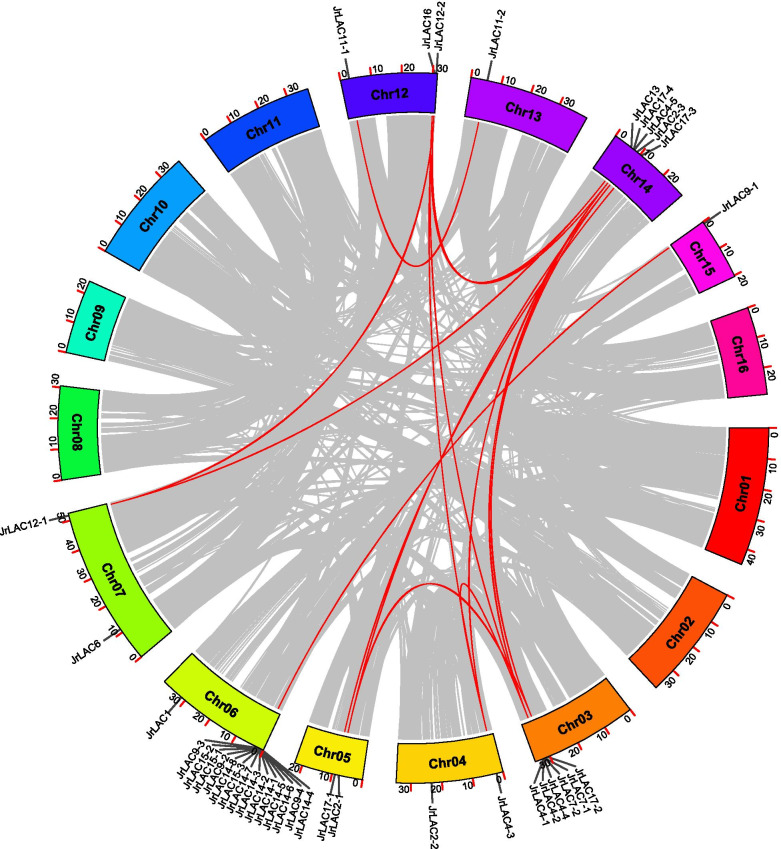
Distribution of segmental duplication-derived LAC genes in the walnut genome. The red lines represent collinear pairs of the JrLAC genes, whereas the gray lines indicate collinear pairs of all walnut genes. The outer circle indicates the location of the JrLACs on each chromosome. The different colored boxes represent the 16 chromosomes of walnuts

The within-genome duplication events of the JrLAC genes were analyzed using MCScan [42]. The results indicated that 16 genes located on chromosomes 3 and 6 are involved in tandem duplication events, and formed eleven gene pairs (Fig. 2; Additional file 1). We also performed a collinearity analysis of LACs to explore the evolutionary relationships of JrLACs. The results showed that 15 syntenic JrLAC gene pairs derived from WGD or segmental duplication were distributed on 9 of 16 chromosomes (Fig. 3; Additional file 2).

**Fig. 4:**
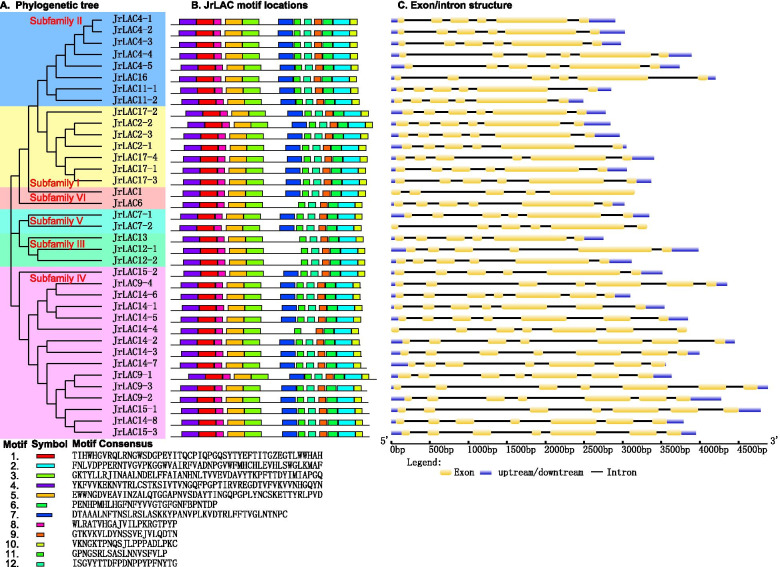
Predicted exon-intron structures of walnut LACs and conserved motifs of walnut LAC protein. **A.** The phylogenetic tree was constructed based on the full-length protein sequences of 37 walnut LAC proteins using MEGA V7.0.21 software. **B.** Location of conserved motifs in walnut JrLAC proteins. Motifs, 1–12, are displayed by differently colored boxes. **C.** Exon-intron structure of walnut LAC genes. The exons and introns are indicated by yellow boxes and single lines, respectively

The exon and intron sequences of the 37 genes were analyzed, and the results showed that subfamilies I, II, III and V all contained six exons and five introns and that all the genes except *JrLAC7–2* had upstream and downstream untranslated regions (UTRs). All the genes in subfamily IV had 7 exons and 6 introns with the exception of except *JrLAC15–2*, which had 6 exons and 5 introns; in addition, all the genes had UTRs except *JrLAC14–4*, which had no upstream or downstream UTR. Subfamily VI had only one copy each of *JrLAC1* and *JrLAC6*, and these genes showed significant differences: *JrLAC1* contained 5 exons and 4 introns and no upstream or downstream UTR, and *JrLAC6* had 6 exons and 5 introns (Fig. 4A and B).

**Fig. 5:**
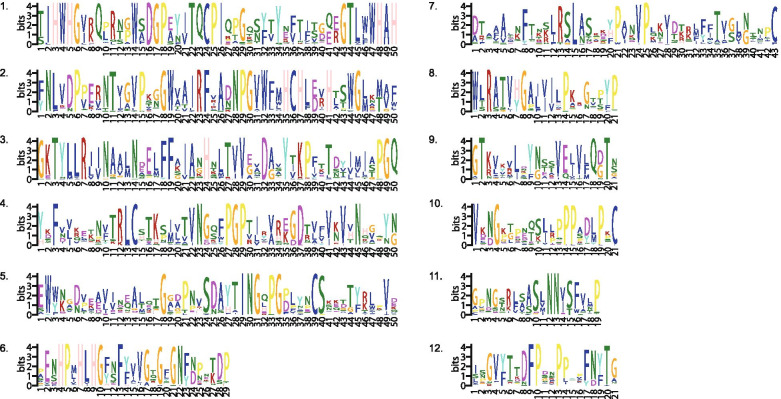
Details of conserved motifs of walnut LAC proteins. The motifs in the JrLACs were identified using Multiple Em for Motif Elicitation (MEME)

An analysis of the amino acid sequences encoded by the genes in this family revealed that all the amino acid sequences contained motifs 1–8 and 11, and motif 10 was missing from three genes in subfamily III and the amino acid sequence encoded by the *JrLAC6* gene in subfamily VI. The amino acid sequence encoded by the *JrLAC14–4* gene in subfamily IV lacked motifs 10 and 9, and the amino acid sequence encoded by the *JrLAC9–4* gene lacked motif 12 (Figs. 4 and 5C).

### Accumulation of lignin in the endocarp at different developmental stages

**Fig. 6:**
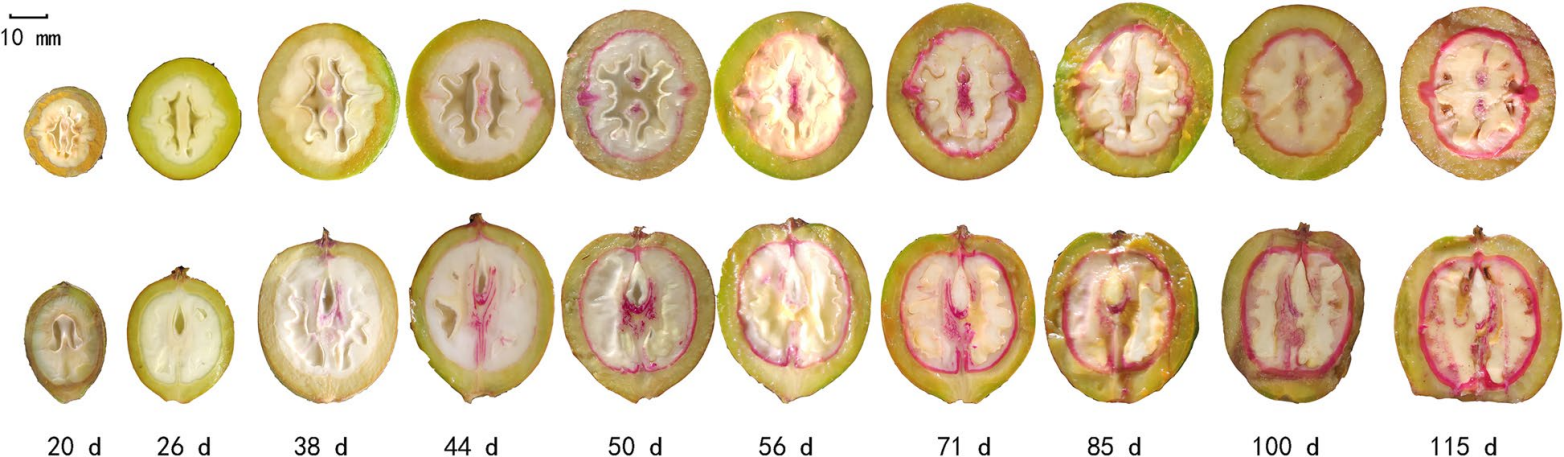
Walnut endocarp lignification process. The upper side shows a transverse cut of the fruit and the lower side shows a longitudinal cut of the fruit. The lignin in endocarp is stained pink based on Wiesner reaction. The fruit shells were not lignified until 38 days after flowering and began to lignify after 44 days after flowering

Walnut is a pseudodrupes; the husk originates from the hairy involucre that surrounds the pistil [2], and the endocarp is gradually lignified during development to form the hard shell. Lignin is stained pink by methanetriol-hydrochloric acid, and thus, the lignification process of walnut was recorded by using this reagent (Fig. 6). The results showed that the fruit was rapidly enlarging, the endocarp cells were in a period of rapid division from 20 days after flowering (DAF) to 38 DAF, and lignification did not occur. At 32 DAF, the placenta inside the fruit appeared pink, which indicated that the start of lignification occurred in this. At 44 DAF, the fruit was no longer enlarged, and some of the tissue at the junction of the endocarp and the husk was stained pink, particularly at the suture, which indicated that the husk began to lignify at this time. However, the inner endocarp remained white, indicating that its lignification proceeded from the outside to the inside. At 50 DAF, the endosperm was still a liquid, and the endocarp was stained in a circle at the junction with the husk, indicating full lignification of the endocarp. Between 50 and 100 DAF, the husk gradually thickened and became increasingly lignified. From 100 to 115 DAF, similar husk staining was observed, and no significant changes occurred, which indicated that the lignification process was nearly complete.

**Fig. 7:**
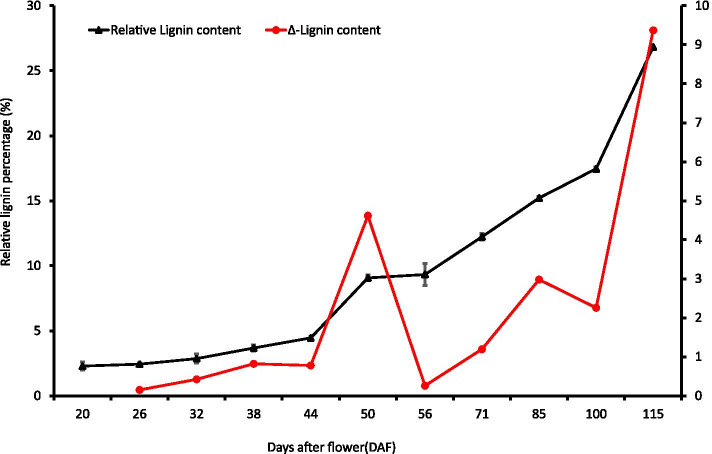
Lignin content of walnut fruit endocarp at different developmental stages. The black line shows the relative lignin percentage of endocarp. The red line shows the changes in the lignin content compared with the previous stage

Further determination of the endocarp lignin content at different developmental stages showed that the lignin content was low (with a relative level below 4%) until 44 DAF. After 44 DAF, the endocarp lignin content increased rapidly, reaching 26.8% by 115 DAF (Fig. 7), and this founding was similar to the results of the Wiesner reaction. Determination of the changes in the Δ-lignin content showed that the rate of increase in the lignin content changed slowly until 44 DAF; however, faster increases were detected from 44 DAF to 50 DAF and from 100 DAF to 115 DAF. This finding indicates that the rapid accumulation of lignin in the walnut endocarp occurred mainly between 44 and 50 DAF and after 100 DAF.

### Expression patterns of JrLAC genes during endocarp lignification

**Fig. 8:**
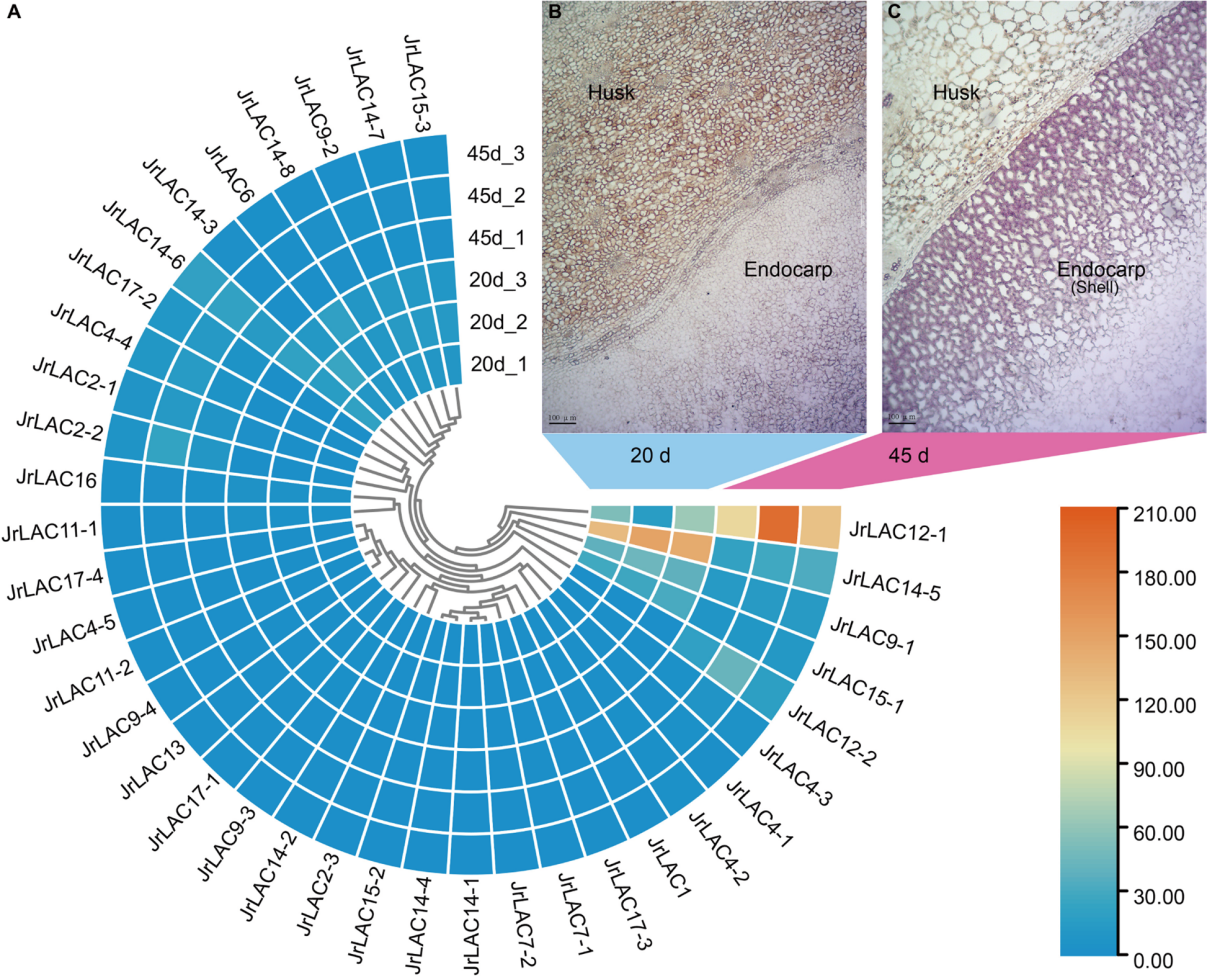
Expression patterns of JrLACs genes. **A** Heat-map depicting the transcriptional activities of 37 JrLAC genes at 20 DAF and 45 DAF. **B** Cross-sectional view of the fruit at the junction of the endocarp and husk at 20 DAF; with the white tissue being the endocarp, and the brown tissue is the mesocarp. **C** Cross-sectional view of the junction of the endocarp and husk at 45 DAF. At this time, the cells were significantly enlarged, the pink tissues in is endocarp, and the cell wall is significantly thickened

We collected samples at 20 and 45 DAF for tissue observation and staining using the methanetriol-hydrochloric acid method (Fig. 8). The results showed that at 20 DAF, the pericarp cells were polygonal and small in size, which indicated that the tissues were in a period of rapid division and had no deposited lignin (Fig. 8B). At 45 DAF, the pericarp cells were significantly enlarged, and exhibited long cell margins at the junction of the mesocarp and endocarp (Fig. 8C). The Wiesner reaction clearly showed that the endocarp cells near the junction began to lignify and the cell walls appeared to thicken, whereas the endocarp cells away from the exocarp had not yet lignified.

The expression level of each *JrLAC* gene was normalized using the FPKM method (Fig. 8A; Additional file 3). Interestingly, the expression level of *JrLAC12–1* during lignification (45 DAF) was higher than that in the nonlignified endocarp (20 DAF). In contrast, the expression of *JrLAC14–5* exhibited the opposite changes. In terms of subfamilies, the total expression of subfamily IV and VI genes was significantly decreased at the lignification stage (45 DAF) than at the unlignified stage (20 DAF) (Additional files 3 and 4). In particular, all subfamily IV genes showed a downregulation trend during lignification. The total expression of the subfamily I, II and III genes was significantly higher at the lignification stage than at the unlignified stage. At both stages, the expression of subfamily III and IV genes was significantly higher than that of genes belonging to the other subfamilies. These results suggest that the *JrLACs* have different functions and that some members are redundant.

### Expression analysis of JrLACs at different developmental endocarp stages

**Fig. 9:**
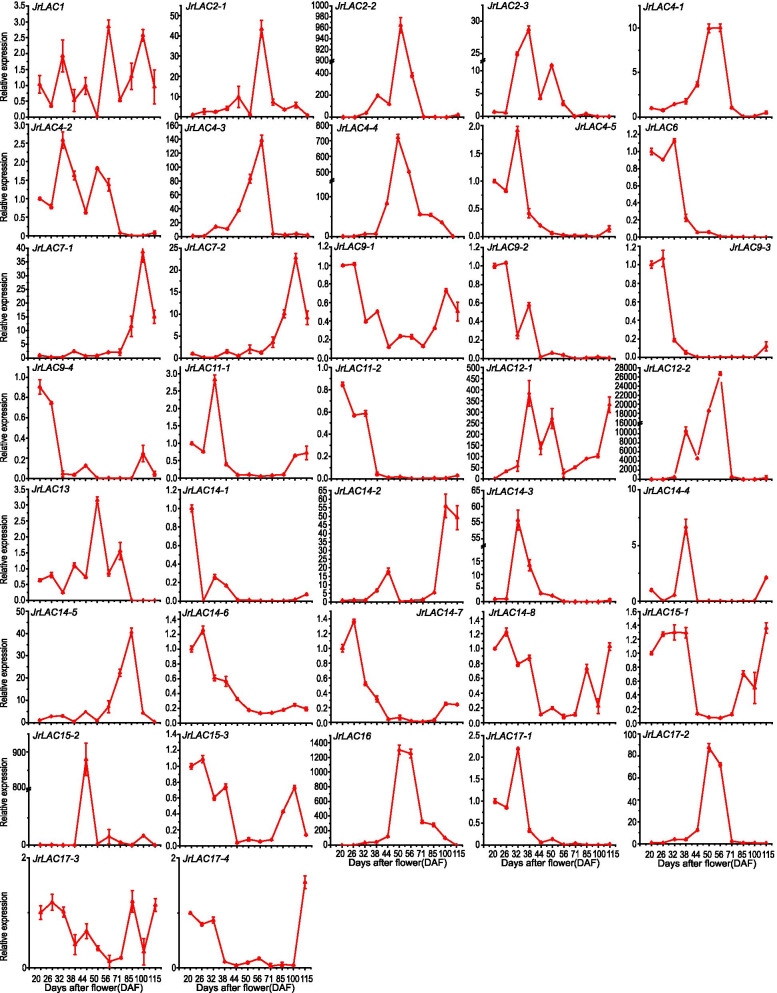
Changes in *JrLACs* expression walnut fruit endocarp at different developmental stages. Each value represents the mean ± SE of three biological replicates

The expression of *JrLACs* during fruit development was further verified by quantitative RT-PCR, and the findings were generally similar to the transcriptome results, but different *JrLACs* showed different expression at different developmental stages (Fig. 9). Among these genes, the expression of *JrLAC4–5*, *JrLAC6*, *JrLAC9–1*, *JrLAC9–2*, *JrLAC9–3*, *JrLAC9–4*, *JrLAC11–2*, *JrLAC14–1*, *JrLAC14–6*, *JrLAC14–7*, *JrLAC15–3*, and *JrLAC17–1* decreased during the lignification of endocarp after 44 DAF, and the expression fold change was small. *JrLAC2–1*, *JrLAC2–2*, *JrLAC2–3*, *JrLAC4–1*, *JrLAC4–2*, *JrLAC4–3*, *JrLAC4–4*, *JrLAC7–1*, *JrLAC7–2*, *JrLAC12–1*, *JrLAC12–2*, *JrLAC13*, *JrLAC14–3*, *JrLAC14–4*, *JrLAC15–2*, *JrLAC16* and *JrLAC17–2* showed a significant increase in gene expression from 38 to 71 DAF followed by decrease, and among these, *JrLAC12–1*, *JrLAC12–2* and *JrLAC16* showed the greats changes. The expression of other genes changed slightly and showed irregular changes. A comparison of the expression period and relative expression of *JrLACs* indicated that different JrLAC genes may play different roles at different periods of shell development. According to the results shown in Figs. 6 and 7, endocarp lignification started at 44 DAF, and the expression of nine genes, *JrLAC2–1*, *JrLAC2–2*, *JrLAC4–1*, *JrLAC4–3*, *JrLAC4–4*, *JrLAC12–1*, *JrLAC12–2*, *JrLAC16* and *JrLAC17–2*, showed a significant increase after 44 DAF. In particular, the expression of *JrLAC12–1*, *JrLAC12–2* and *JrLAC16* exhibited extremely significant expression changes; thus, it can be hypothesized that *JrLAC12–1*, *JrLAC12–2* and *JrLAC16* may be involved in the process of shell lignin accumulation. The relative expression of *JrLAC12–1* increased starting from 32 DAF, peaked at 38 DAF, and remained at a high level until 50 DAF (Fig. 9). In addition, the expression level of *JrLAC12–1* remained high from 85 to 100 DAF, which is also the period when the shell appeared and lignin was deposited in large quantities (Figs. 6 and 7); thus, it is assumed that *JrLAC12–1* may play a key role in the process of shell lignin accumulation.

### Expression analysis of JrLACs from different subfamilies in different walnut tissues

**Fig. 10:**
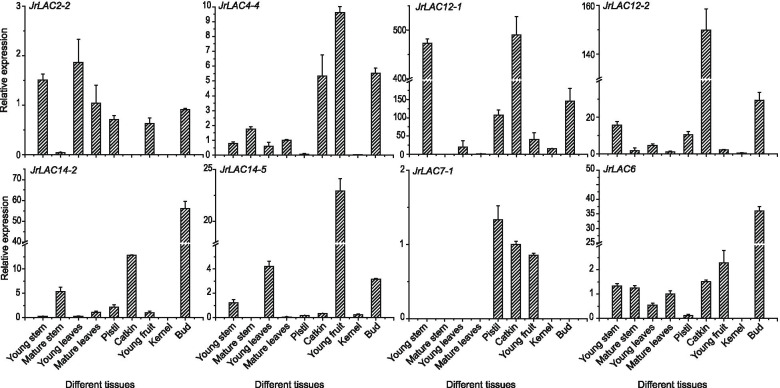
Changes in *JrLAC* expression in different tissues. Each value represents the mean ± SE of six biological replicates. Subfamily I: *JrLAC2–2*; Subfamily II: *JrLAC4–4*; Subfamily III: *JrLAC12–1* and *JrLAC12–2*; Subfamily IV: *JrLAC14–2* and *JrLAC14–5*, Subfamily V: *JrLAC7–1*; Subfamily VI: *JrLAC6*

The specific expression of *JrLACs* from different subfamilies in different tissues of walnut was further analyzed, and eight genes in six subfamilies showed significantly different expression patterns in each tissue (Fig. 10). *JrLAC2–2* in subfamily I is expressed at relatively high levels in young stems and leaves but exhibits low or no expression in mature stems, catkins and kernels; *JrLAC4–4* in subfamily II is highly expressed in catkins, young fruits, and buds and is found at low or no expression in pistils and kernels; *JrLAC12–1* and *JrLAC12–2* in subfamily III showed similar trends, i.e., high expression in young stems, catkins, pistils and buds and low expression in mature stems and mature leaves, in particular, young woody stems are the site of rapid lignification of plant, and *JrLAC12–1* exhibits higher expression in this tissue; in subfamily IV, *JrLAC14–2* is highly expressed in catkins and buds, and *JrLAC14–5* is highly expressed in young leaves and young fruits and exhibits lower expression in mature stems and mature leaves; *JrLAC7–1* in subfamily V is expressed only in pistils, catkins and young fruits; *JrLAC6* in subfamily VI is highly expressed in buds and shows low expression in pistils and kernels. Most *JrLACs* were found to be expressed at low levels in mature tissues and at high levels in young tissues. The expression of genes was higher in young tissues, where lignin is biosynthesized and deposited in large quantities (Additional file 8), whereas their expression was lower in mature organs because these the organs had completed development and no longer needed to accumulate lignin. Because the expression of the *JrLAC* genes differed among tissues, it can be assumed that these genes have different functions in the lignification process.

### Correlation between the lignin content and JrLAC expression

To further verify the relationship between lignin synthesis and LAC genes, we conducted a correlation analysis between the lignin content of the endocarp and the expression of the 37 genes at different developmental stages (Table 2), and the results showed a significant positive correlation between *JrLAC12–1* and Δ-lignin content, whereas no correlation was found between the other genes and Δ-lignin content. Although the expression of genes such as *JrLAC4–2*, *JrLAC6*, *JrLAC7–1*, *JrLAC7–2*, *JrLAC9–2* and *JrLAC14–2* was significantly positively or negatively correlated with the lignin content, expression trend of these genes is similar or opposite to the cumulative amount of lignin because the lignin is irreversible during the cumulative process. This result further suggests that *JrLAC12–1* is involved in the deposition of lignin in the endocarp.

**Table 2:** Correlation analysis between genes expression and lignin content

Genes	Lignin content	Δ-Lignin content
*LAC1*	0.231	−0.236
*LAC2–1*	0.045	−0.366
*LAC2–2*	−0.080	0.141
*LAC2–3*	−0.470	−0.229
*LAC4–1*	−0.115	−0.083
*LAC4–2*	−0.624^a^	−0.321
*LAC4–3*	−0.036	−0.158
*LAC4–4*	0.024	0.064
*LAC4–5*	−0.595	−0.334
*LAC6*	−0.662^a^	−0.406
*LAC7–1*	0.647^a^	0.323
*LAC7–2*	0.687^a^	0.349
*LAC9–1*	−0.204	−0.008
*LAC9–2*	−0.646^a^	−0.372
*LAC9–3*	−0.471	−0.227
*LAC9–4*	−0.390	−0.229
*LAC11–1*	−0.282	−0.117
*LAC11–2*	−0.572	−0.362
*LAC12–1*	0.303	0.633^a^
*LAC12–2*	−0.077	−0.122
*LAC13*	−0.282	−0.034
*LAC14–1*	−0.389	−0.068
*LAC14–2*	0.715^a^	0.564
*LAC14–3*	−0.381	−0.267
*LAC14–4*	−0.102	0.139
*LAC14–5*	0.251	−0.092
*LAC14–6*	−0.660^a^	−0.397
*LAC14–7*	−0.491	−0.243
*LAC14–8*	−0.136	0.235
*LAC15–1*	−0.082	0.214
*LAC15–2*	−0.221	−0.199
*LAC15–3*	−0.450	−0.328
*LAC16*	0.078	−0.011
*LAC17–1*	−0.579	−0.344
*LAC17–2*	−0.021	0.021
*LAC17–3*	−0.033	0.276
*LAC17–4*	0.172	0.544

^a^Correlations were considered significant at the 0.05 levels

## Discussion

According to their oxidation characteristics, LACs are divided into two groups: low-redox-potential and high-redox-potential enzymes [43]; therefore, LACs can catalyze both anabolic and catabolic reactions. In fungi, LACs are involved in catabolism and can degrade lignin and humus [44], whereas in higher plants or insects, LAC catalyzes anabolic reactions involved in various morphogenesis processes, such as lignin synthesis [45], cuticle formation [46], and flavonoid synthesis [47]. Therefore, this finding indicates that the LAC gene family may differ significantly among species of different taxa. In fungi, the LAC gene family is divided into five clusters, and LACs within different clusters can be induced by different substrates [48]. In higher plants, the LAC genes within different species vary, and the LAC gene family of each plant is usually divided into six subfamilies based on protein sequence characteristics, such as those of soybean [36], *S. viridis* [37], and sweet cherry [40]. However, in *C. sinensis* and *Brassica napus*, the LAC gene family is divided into seven and five subfamilies, respectively [35, 49]. Differences in the degree of expansion of LAC genes have been found within different species, such as in subfamilies III and II in *B. napus* [49], subfamily V in *S. viridis* [37], soybean [36] and pear [31], and subfamily I in *C. sinensis* [35]. In sweet cherry [40], which is also a type of drupe, the LAC gene family is significantly expanded in subfamilies II and IV, similar to the result obtained for walnut in the present study. Previous studies have also shown that some of the walnut LAC genes in subfamily II or IV are distributed as chromosomal gene clusters, and 11 pairs of genes in subfamilies II, IV and V show tandem duplication events. The tandem duplication of these LAC genes in the walnut genome may be the main reason for the expansion of subfamilies II and IV. In addition, 15 pairs of genes with collinearity belonged to subfamilies I, II, III and IV, and it is speculated that these collinear pairs of genes might have similar functions.

Xiao *et al*. found that most of the genes that are highly expressed in lignified tissues, such as roots and stems of *B. napus,* belong to LAC subfamilies I, II and III [49]. In the C4 model grass *S. viridis*, *SvLAC52, SvLAC15* and *SvLAC9* (*AtLAC2* or *LAC17* homologs) in subfamily I and *SvLAC50* (homolog of *AtLAC16*) in subfamily II are also highly expressed in the transitional and maturation zones, where lignin biosynthesis-related genes are active [37]. In soybean, the genes *GMLAC2* and *GmLAC9* (*AtLAC*4 homologs) in subfamily II and *GmLAC8* (*AtLAC12* homolog) in subfamily III are highly expressed in stems, whereas the 11 genes showing constitutive expression in multiple tissues all belong to subfamily V [36]. In pear, *PbLAC1* (*AtLAC2* homolog), *LAC38* and *LAC29* (*AtLAC17* homologs) in subfamily I and *PbLAC5*, *PbLAC36*, and *PbLAC6* (*AtLAC4* homologs) in subfamily II are highly expressed at the stage showing peak lignin content in the pulp, whereas *PbLAC10*, *PbLAC11* and *PbLAC39* (*AtLAC15* homologs) in subfamily IV and *PbLAC20* (*AtLAC14* homolog) in subfamily IV exhibit the exact opposite results. The LAC genes associated with lignin synthesis in previous studies belong mostly to subfamilies I, II and IV. *AtLAC4* in subfamily II in Arabidopsis may be involved in the synthesis of lignin and secondary walls. *AtLAC4* is strongly expressed in vascular tissues and specifically in leaf hydathodes, and *AtLAC4* overexpressing plants show a dwarf phenotype and have shorter leaf petioles, both of which are effects caused by increase in the lignin content and secondary wall thickness [50]. The expression of *JrLAC4–1*, *JrLAC4–2*, *JrLAC4–3*, *JrLAC4–4* and *JrLAC16* in subfamily II in walnut, particularly the latter, increased significantly from 44 to 71 DAF. It can be speculated that LAC genes in subfamily II also assume important functions during endocarp lignification in walnut. In addition, the expression patterns of the six subfamilies differed between the unlignified and lignified stages of the endocarp, as demonstrated by an increase in the total expression of subfamilies I, II, III and a decrease in the total expression in subfamilies IV and VI. Based on previous studies and the results of this study, six subfamilies have different functions in endocarp lignification, and the division of labor between these subfamilies is different in walnut.

The final step in lignin biosynthesis is the polymerization of lignin monomers by dehydrogenation in response to enzymes such as POD, LAC and polyphenol oxidase (PPO) [51]. Previous studies have concluded that PODs use hydrogen peroxide (H_2_O_2_) to oxidize their substrates [52]. In contrast to PODs, LACs consume O_2_ instead of H_2_O_2_ to oxidize monolignols. LACs of a variety of species are expressed in lignifying cells [53]. The lignification of cells from different parts of the plant may proceed in different manners, depending on whether the lignin is local or widespread. Casparian strip formation requires PODs but not LACs [54]. The walnut shell consists of sclereids formed by the thickening and lignification of the secondary walls of the parenchyma in the endocarp [13]. Our previous study found that shell formation is accompanied by increases in the lignin, cellulose and phenolic content, no mark change in PPO activity and POD activity, and that POD activity is negatively correlated with lignin accumulation [12]. In this study, the expression pattern of LAC genes during lignification was further analyzed. Subfamily III genes, such as *JrLAC12–1* and *JrLAC12–2* were highly expressed during the lignification stage; in particular, the expression of *JrLAC12–1* showed a significant positive correlation with the change in the lignin content. Consistent with the results obtained for walnut shell sclereids, sclereids in pear pulp also show high LAC expression during the developmentally critical stage of formation [31], and the silencing of three LAC genes reduces the lignin content and sclereid number in pear fruit [55]. Therefore, LACs may play a key role in the lignification of walnut shells.

Previous studies found that in poplar, *PtoLAC14* is mainly expressed in lignified tissues in the vascular bundles, such as the stem xylem, and that the overexpression of *PtoLAC14* resulted in smaller cells with secondary wall thickening and an increased lignin content [56]. In *Arabidopsis*, *AtLAC4* in interfascicular fibers and seed coat columella, *AtLAC15* in seed coat cell walls, *AtLAC14* in vascular tissues, such as roots, stems, leaves, petals and leaves but not in siliques (the fruit of *Arabidopsis*), are uniquely expressed [34]. In contrast, *AtLAC12* is expressed in all tissues of *Arabidopsis*, particularly in the replum and abscission zone of the siliques. In this study, high expression of *JrLAC14–1*, *JrLAC14–3*, *JrLAC14–4*, *JrLAC14–6* and *JrLAC14–7* were observed in young fruits, which was presumed to be mainly related to vascular tissue formation at the early stages of young fruit development period. *JrLAC12–1*, *JrLAC12–2* and *JrLAC6* are highly expressed in young stems and buds, and the changes in the expression of *JrLAC12–1* during shell development were consistent with the changes in lignin deposition, which suggested that *JrLAC12–1* is involved in tissue formation and is a key factor in the lignification of sclereids in the shell. Therefore, the regulatory mechanism of *JrLAC12–1* in walnut pericarp development needs to be further investigated.

## Conclusion

Thirty-seven LAC genes were identified in the walnut genome, are distributed on nine chromosomes and can be divided into six subfamilies. During walnut endocarp development, different *JrLACs* show significantly different expression trends, and *JrLAC12–1*, *JrLAC12–2* and *JrLAC16* may be involved in endocarp lignification. Changes in walnut *JrLAC12–1* gene expression were found to be positively correlated with Δ-lignin in the shell, which suggests that *JrLAC12–1* plays a key role during lignin accumulation in endocarp lignification.

## Methods

### Plant and materials

The material used in this study was collected in 2016–2017 from the Experimental Field of Hebei Agricultural University (EFHAU) (EFHAU; 38°48′N, 115°24′E), China. No special permission was necessary to collect such samples. The walnut cultivar ‘Zanmei’ in the full fruit-bearing period was selected as the test material. The plants were pruned when necessary and received standard fertilization. Fresh fruit of ‘Zanmei’ were collected every 5 days starting at the early stage of fruit growth and every 14 days after the shell appeared from 20 DAF until 115 DAF. Thirty samples of walnut fruit at each stage were randomly collected from all four cardinal directions on the ‘Zanmei’ trees. After the fresh fruit cut, part of the pericarp was frozen with liquid nitrogen and frozen to −80 °C for transcriptome sequencing, gene expression analysis and lignin content determination, and the other part was cut into ≈5 mm^3^ cubes and fixed in 2.5% (v/v) glutaraldehyde and 3% (v/v) polyformaldehyde in phosphate-buffered saline [PBS; 4 mM sodium phosphate (pH 7.2), 200 mM sodium chloride (NaCl)] [13]. Tissues (root, young stem, mature stem, young leaf, mature leaf, pistil, catkin, young fruit, kernel and bud tissues) were collected separately from field-grown trees at EFHAU, frozen with liquid nitrogen and frozen to −80 °C for organ-specific expression analyses.

### Identification of the LAC gene family and bioinformatic analysis

The sequences of *Arabidopsis* LAC proteins were downloaded from the UniProt protein database (http://www.uniprot.org). The hidden Markov model was constructed using HMMER 3.1b software and the *Arabidopsis* LAC family protein sequences (Additional file 7) in the UniProt database, and the protein sequences of walnut were downloaded from GenBank (accession GCA_001411555.2). The conserved domains of the proteins were analyzed with the Pfam protein family database (http://pfam.org/) [57] of the European Institute of Bioinformatics using the Gene Structure Display Server (GSDS, http://gsds.cbi.pku.edu.cn/) [58] and MEME (http://meme-suite.org/) for the walnut LAC family. The phylogenetic tree of the JrLAC and AtLAC gene families was constructed with the maximum likelihood method using MEGA 7.0 software with a bootstrap value of 1000.

The physical locations of the JrLAC genes were obtained from the genome annotation information (gff3) of *J. regia*, and the JrLAC genes were mapped to the walnut chromosomes.

The duplication events of the JrLAC genes were calculated using MCScanX software [42] and then, visualized using TBtools software [59]. The CDSs and protein sequences of collinear gene pairs were compared, and the ratio of nonsynonymous substitution to synonymous substitution (Ka/Ks) was calculated using TBtools software [59]. The figure was processed using Adobe Illustrator software.

### Analysis and validation of RNA-Seq data

The endocarp of walnut collected at 20 DAF (unlignified stage) and 45 DAF (lignification stage) was selected for transcriptome sequencing, and three replicates were used in the analysis. The sequence data were deposited in GSA (https://bigd.big.ac.cn/gsa/browse/CRA005050) [60]. Using RNA-Seq, we explored the gene expression patterns of the JrLAC gene family in the endocarp at the unlignified and lignification stages.

### Gene expression analysis by quantitative RT-PCR

RNA isolation from different tissues and the endocarp at different developmental stages was performed using an isolation kit (product number: DP441; Tiangen, Beijing, China). The isolated RNA was solubilized in diethyl phosphorocyanidate (DEPC)-treated RNase-free water and treated with DNase. Pure RNA was used for cDNA synthesis with a commercial kit (product number: RR047A; Tiangen, Beijing, China) according to the manufacturer’s guidelines. The resulting cDNA was diluted 9-fold and stored at −20 °C for subsequent RT-PCR and qRT-PCR assays.

Specific primers for *JrLAC* genes expression quantification were designed using Primer Premier 5.0 software. qRT-PCR experiments were performed using an FX96 Touch™ Real-Time PCR Detection System (Bio-Rad) in conjunction with TransStart Top Green qPCR SuperMix (product number: AQ131; TransGen Biotech, Beijing China). Primers (Additional file 8) were used to analyze the *JrLACs* transcripts. *JrACT2* (NCBI reference: XM_018972062.1) was used as the reference gene to calculate the relative fold differences based on the comparative cycle threshold (2^-ΔΔct^) [61]. The PCR mixture (20 μL) consisted of 10 μL of 2 × TransStart Top Green qPCR SuperMix, 1 μL of each primer (10 μmol/L), 1 μL of diluted cDNA and 7 μL of ddH_2_O. The following conditions were used for RT-PCR: 95 °C for 15 min; 40 cycles of 95 °C for 10 s, 60 °C for 30 s, and 72 °C for 45 s; and a final extension at 72 °C for 10 min. Three experiments (biological replicates) were performed for each sample.

### Preparation of specimen for light microscopy

After extensive washing in PBS, the samples of fixed endocarp were processed as follows: sequentially dehydrated at ambient temperature in 70, 85, 95, and 100% ethanol (1 h for each step); vitrified with a gradient from 100% ethanol to 100% xylene; and infiltrated with and embedded in paraffin [13]. Eight-micron sections were prepared using a microtome (KEDEE KD-225) mounted on adhesive microscopic slides, stained with 2% phloroglucinol in 95% ethanol solution for 5 min and mounted in 6 M HCl. Subsequently, the sections were examined with an Olympus (Tokyo, Japan) microscope (BH-2).

### Lignin deposition and determination

The fruit was longitudinally and transversely cut into halves for the Wiesner test (Lin *et al*., 2002). Each part was treated with 2% phloroglucinol in a 95% ethanol solution for 5 min and mounted in 6 M HCl to examine the presence of the cinnamaldehyde groups of the lignin [12].

The endocarps of walnut fruit were chopped into small pieces, frozen in liquid N_2_, and ground to a fine powder with a grinder. This lyophilized powder was used for lignin content determination, which was carried out as described by Zhao *et al* [12]. The change in the lignin content (Δ-lignin content) was calculated by subtracting the lignin content in the previous period from the lignin content in the current period.

### Statistical analysis

The experiments were established in accordance with a completely randomized design. The data are shown as the means ± standard errors (SEs) of 3 or 6 independent biological replicates. The data were analyzed with SPSS version 20.0 (IBM Corp., Armonk, NY, USA) and Excel 2016 software (Microsoft Corp., Redmond, WA, USA).

## Supplementary Information

**Additional file 1: Table S1.** Tandem genes of the JrLAC gene family.

**Additional file 2: Table S2.** Collinearity data of the JrLAC gene family*.*

**Additional file 3: Table S3.** Expression of *JrLAC* genes at the unlignified and lignified stages.

**Additional file 4: Figure S1.** Heatmap of JrLAC subfamily expression.

**Additional file 5. **LAC protein sequences of *Arabidopsis.*

**Additional file 6. **LAC protein Sequences of *J. regia.*

**Additional file 7.** Primers.

**Additional file 8.** Relative lignin percentage of different tissues.

**Additional file 9.** Original photographs.

## Data Availability

The RNA-Seq datasets are available in the National Genomics Data Center, China National Center for Bioinformation (https://bigd.big.ac.cn/gsa/browse/CRA005050; accession number: CRA005050). The CDS and genome sequences of LACs in walnut were retrieved from the whole walnut genome database (accession GCA_001411555.2) in NCBI. All data and materials are presented in the main paper and additional file.

## Abbreviations

LAC: Laccase
PPO: Polyphenol oxidase
POD: Peroxidase
