# Supplementary figures and images for: The role of JrLACs in the lignification of walnut endocarp
Source: BMC Plant Biol. 2021 Nov 3;21:511. doi: 10.1186/s12870-021-03280-3 (PMC8565057; doi:10.1186/s12870-021-03280-3)

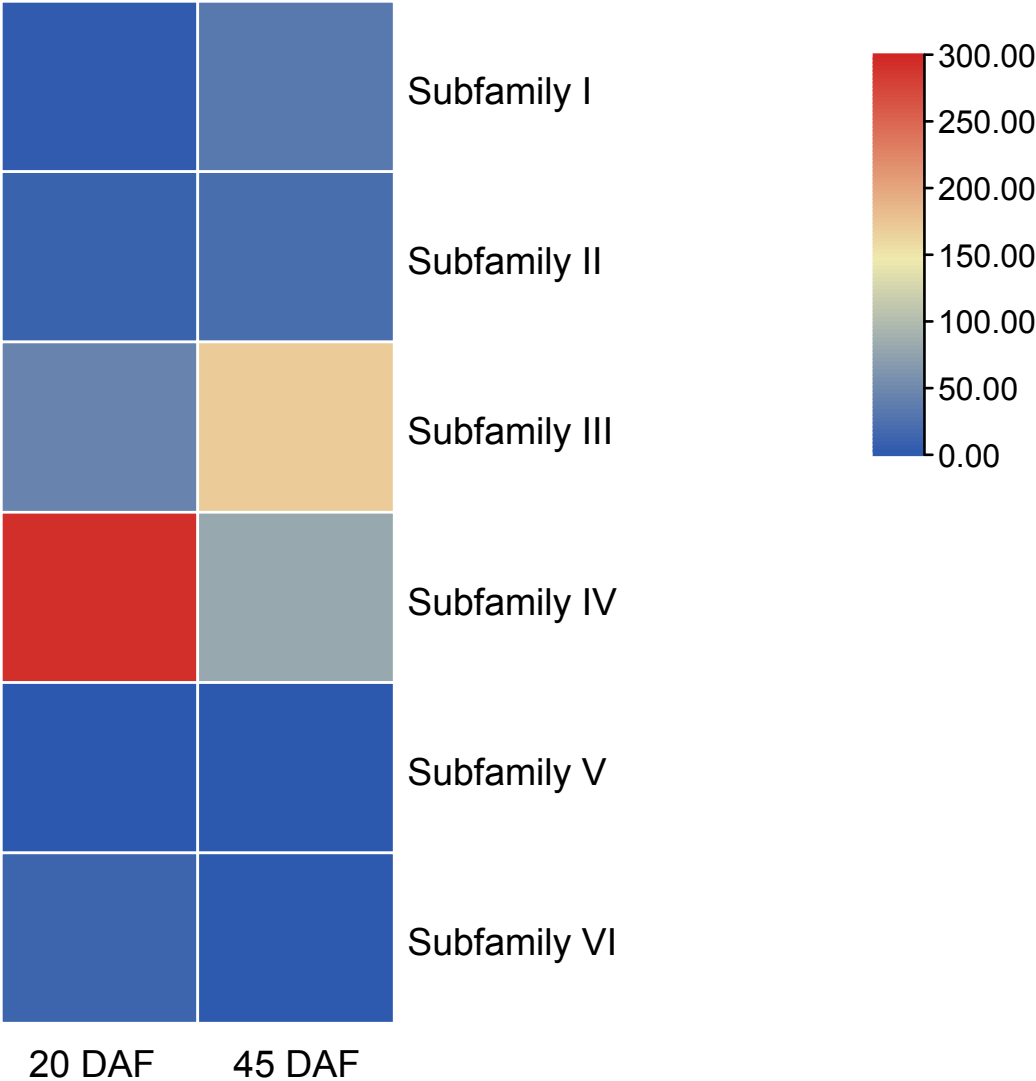

Supplement: Supplementary file 4 — Additional file 4: Figure S1. Heatmap of JrLAC subfamily expression. [file 12870_2021_3280_MOESM4_ESM.pdf]

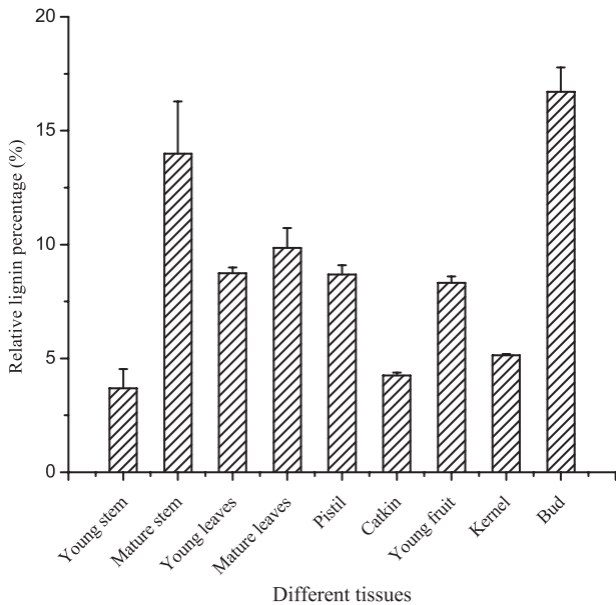

Supplement: Supplementary file 8 — Additional file 8. Relative lignin percentage of different tissues. [file 12870_2021_3280_MOESM8_ESM.pdf]
