# Supplementary material for: The role of JrLACs in the lignification of walnut endocarp
Source: BMC Plant Biol. 2021 Nov 3;21:511. doi: 10.1186/s12870-021-03280-3 (PMC8565057; doi:10.1186/s12870-021-03280-3)
Supplement: Supplementary file 9 — Additional file 9. Original photographs. [file 12870_2021_3280_MOESM9_ESM.docx]

Original pictures of different developmental stages

1. Lignin deposition


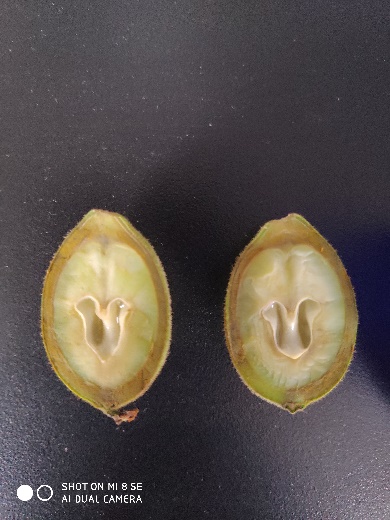

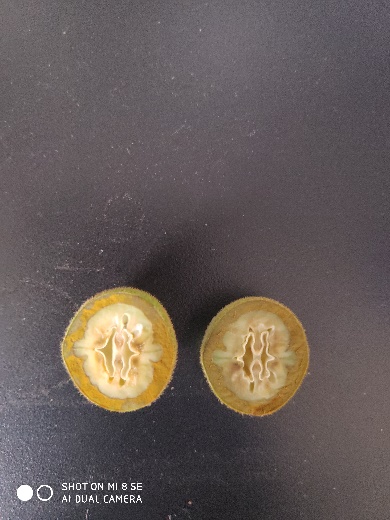


20 DAF


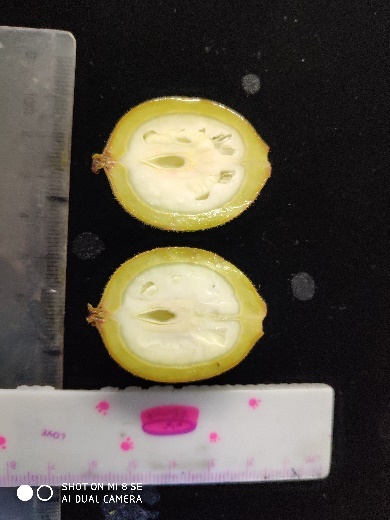

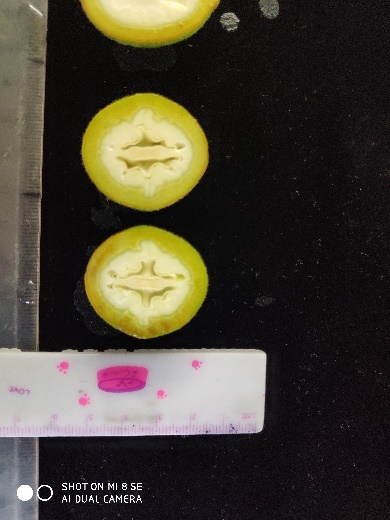


26 DAF


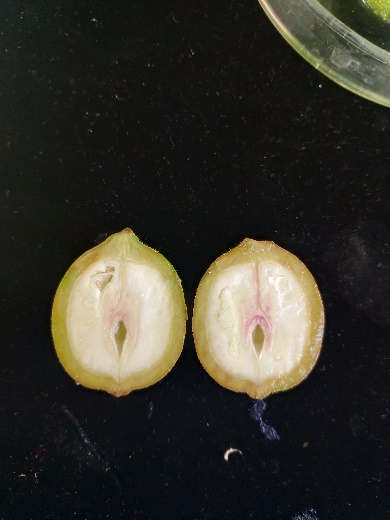


32DAF


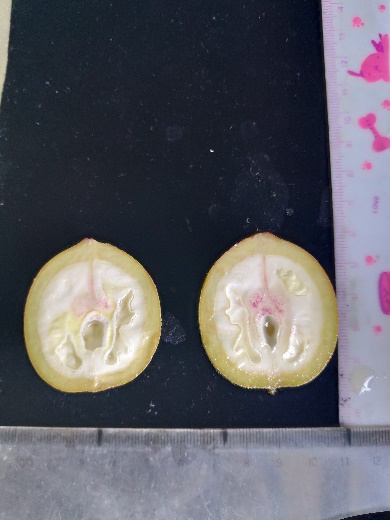

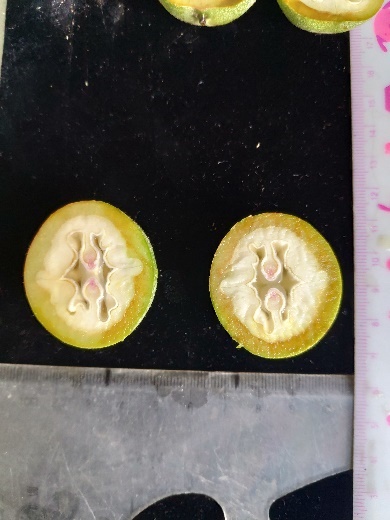


38 DAF


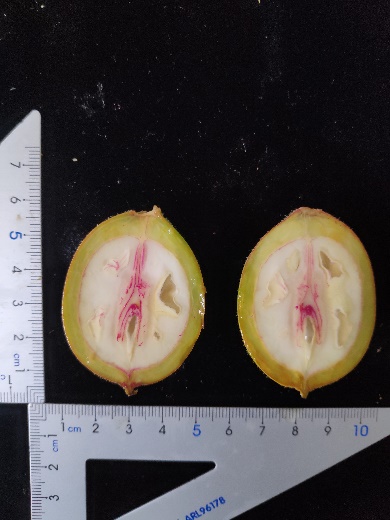

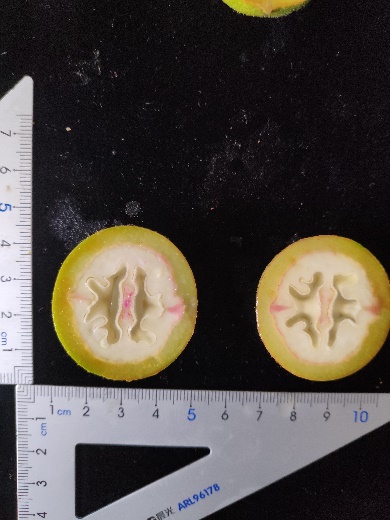


44 DAF


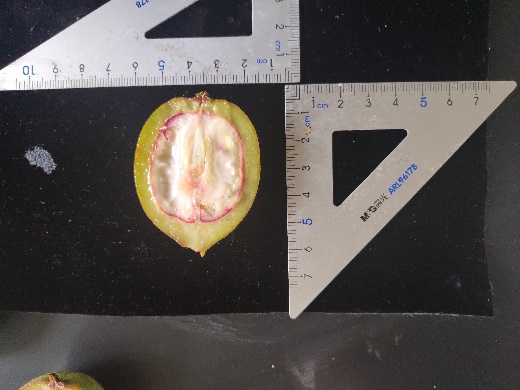

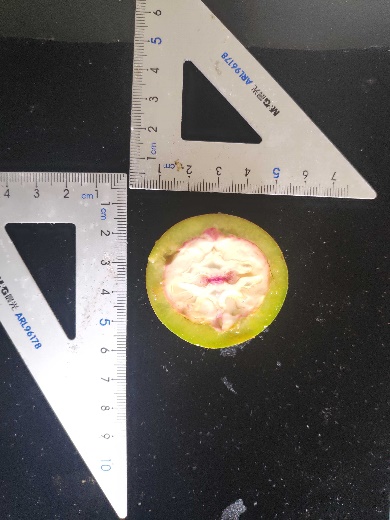


56 DAF


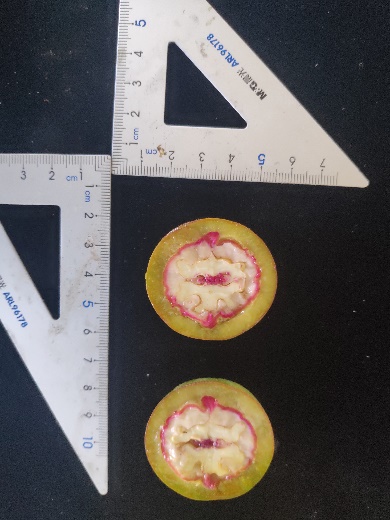

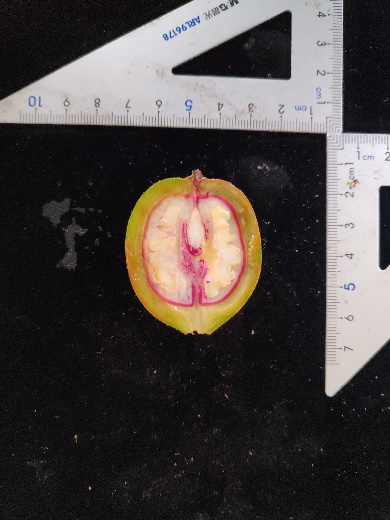


71DAF


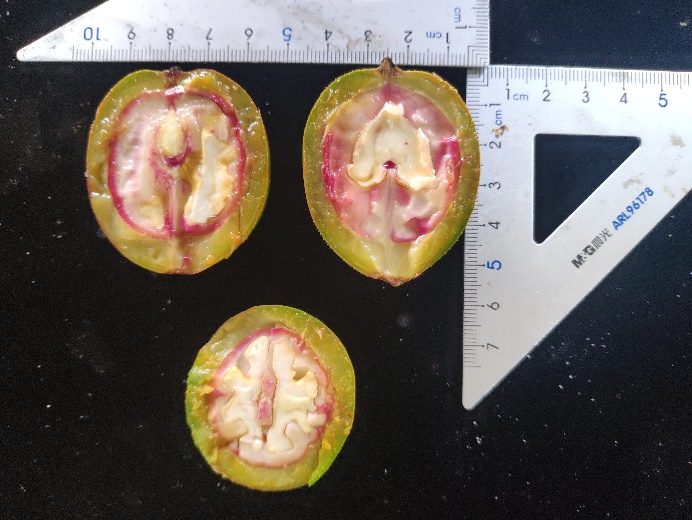


85DAF


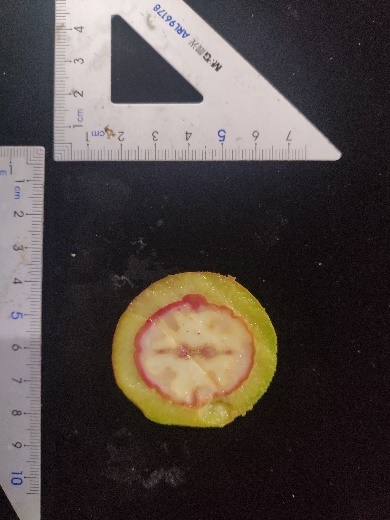

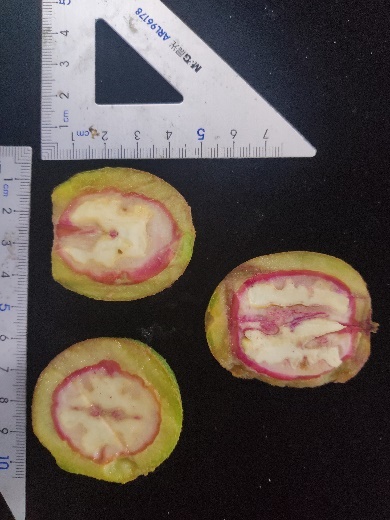


100DAF


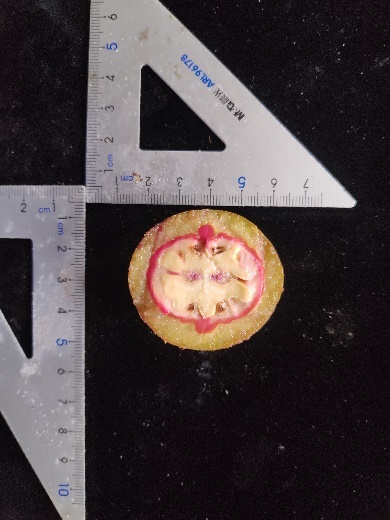

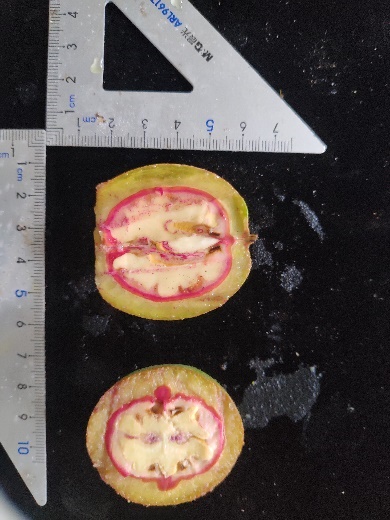


115DAF

1. Sections


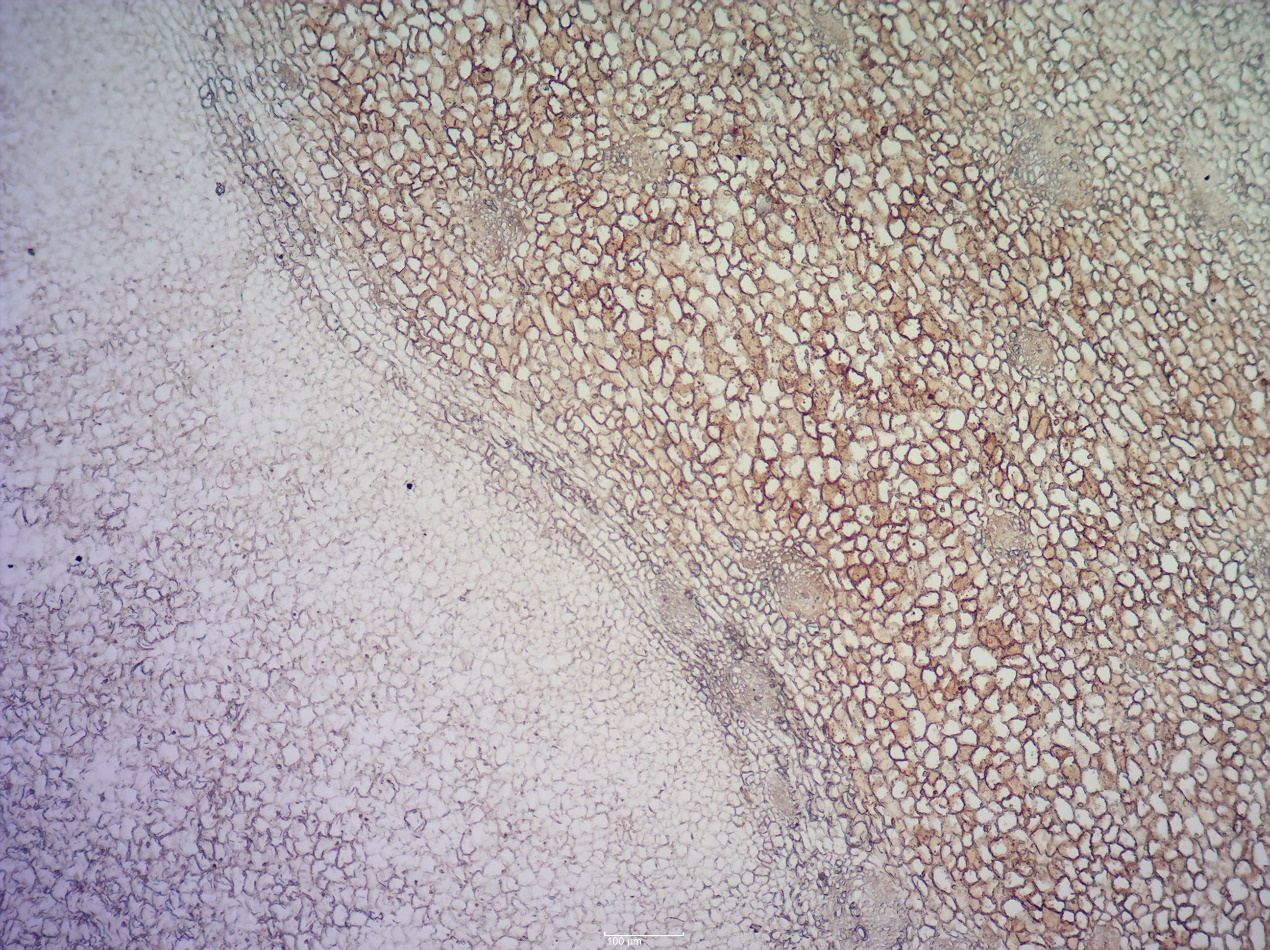


20 DAF


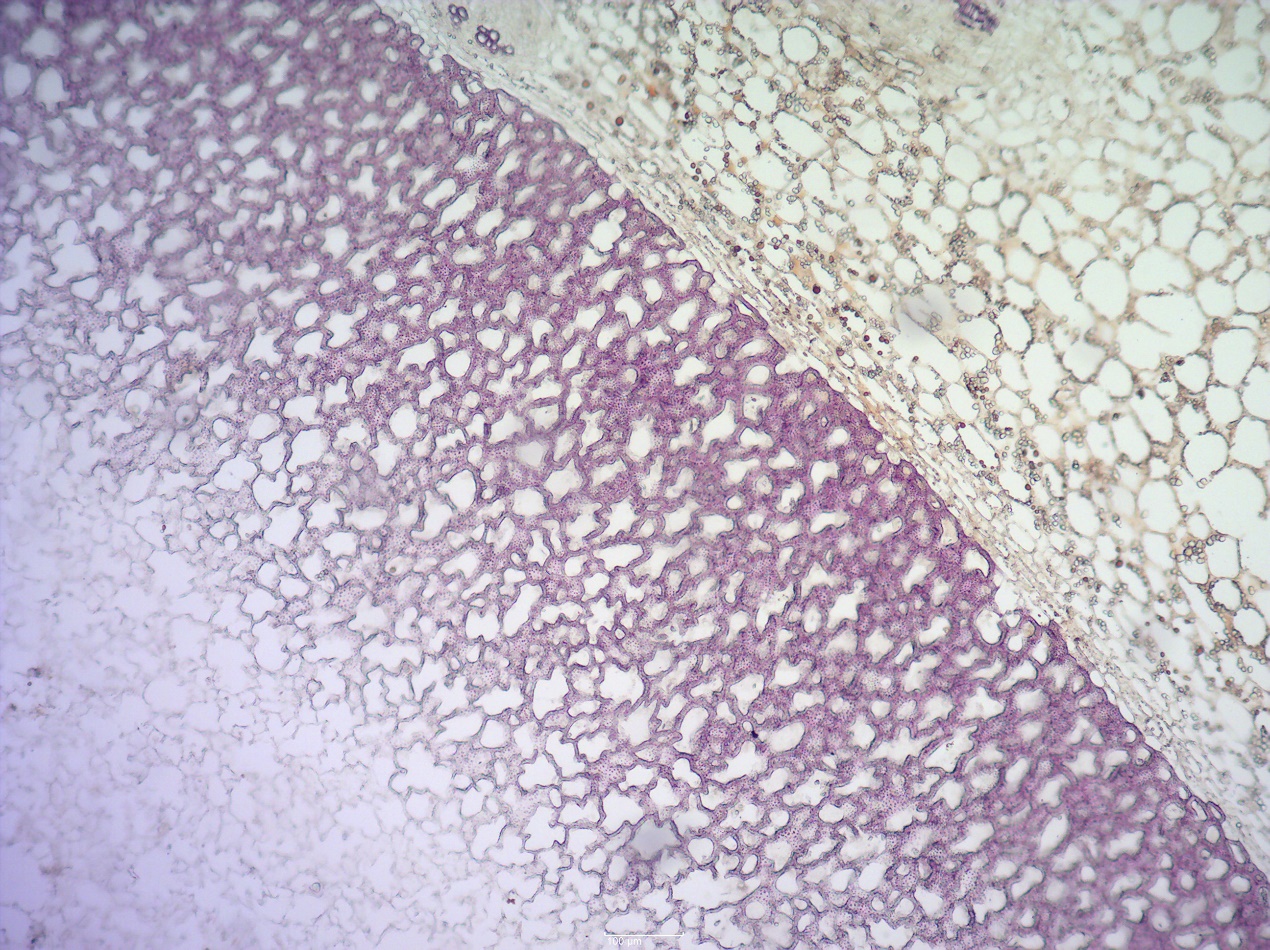


45 DAF
